# Piperine reverses colistin resistance in multidrug resistant Gram-negative pathogens by membrane disruption and ROS damage

**DOI:** 10.1080/21505594.2026.2691333

**Published:** 2026-06-30

**Authors:** Junkai Zhang, Qiange Liu, Haotian Shao, Mengjing Feng, Zibo Li, Yameng Feng, Hua Wu, Dandan He, Yajun Zhai, Gong-Zheng Hu

**Affiliations:** a Department of Veterinary Medicine, Henan Agricultural University, Zhengzhou, Henan, PR China; b Department of Veterinary Drug, Shangqiu Meilan Biological Engineering Co., LTD, Shangqiu, Henan, PR China; c Ministry of Education Key Laboratory for Animal Pathogens and Biosafety, Henan Agricultural University, Zhengzhou, Henan, PR China; d Key Laboratory of Quality and Safety Control of Poultry Products (Zhengzhou), Ministry of Agriculture and Rural Affairs, Zhengzhou, Henan, PR China

**Keywords:** Piperine, colistin, MCR-1, antibiotic resistance, synergistic mechanism

## Abstract

The global spread of colistin (COL) resistance, particularly mediated by *mcr-1*, threatens the efficacy of this last-line antibiotic against multidrug-resistant (MDR) Gram-negative pathogens. This development underscores the urgency of developing innovative therapeutic strategies to counteract antimicrobial resistance mechanisms. This study demonstrates that piperine (PIP), a natural alkaloid, functions as a potent adjuvant that restores COL efficacy through a multi-target mechanism. The combination of PIP and COL exhibits significant synergism against clinically relevant pathogens, including *mcr-1*-positive strains (FICI: 0.07–0.281), and was not susceptible to the development of drug resistance. Additionally, this combination effectively inhibits and eradicates biofilms. Mechanism studies and omics analysis confirmed that PIP combined with COL could cause bacterial membrane disruption, proton motive force (PMF) disruption, efflux pumps inhibition, resulting in accumulation of bacterial reactive oxygen species (ROS) and finally cell death. Furthermore, PIP suppresses MCR-1 through dual suppression of gene expression and protein function. The therapeutic potential was confirmed in murine infection models, where the combination significantly improved survival rates, reduced bacterial loads, and attenuated inflammatory responses. This study provides the first comprehensive elucidation of the multi-target synergy between COL and PIP, offering a promising therapeutic strategy that simultaneously overcomes existing resistance and impedes resistance development.

## GRAPHICAL ABSTRACT

**Figure:**
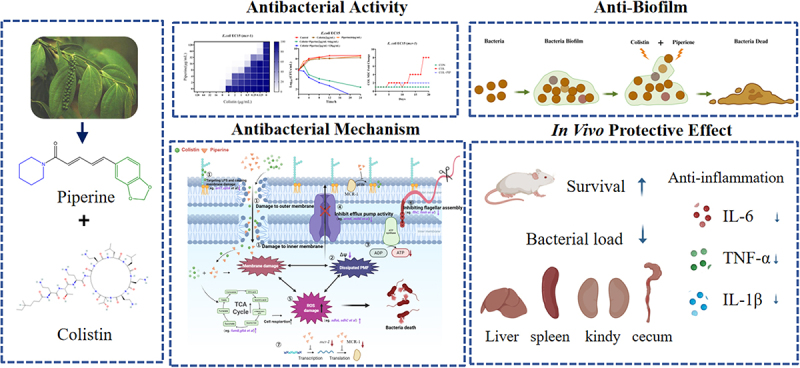

## Introduction

The global spread of antimicrobial resistance (AMR) represents one of the most pressing challenges in modern healthcare, particularly affecting the treatment of Gram-negative bacterial infections [1,2]. Pathogens such as *Escherichia coli* (*E. coli*), *Salmonella*, *Klebsiella pneumoniae* (*K. pneumoniae*), and *Acinetobacter baumannii* (*A. baumannii*) have developed sophisticated resistance mechanisms that severely limit therapeutic options [3–5]. In this critical context, polymyxins, specifically colistin (COL), have reemerged as last-resort therapeutics against multidrug-resistant (MDR) Gram-negative bacteria infections. COL exerts its bactericidal activity through electrostatic interactions with lipopolysaccharides (LPS) in the outer membrane, disrupting membrane integrity and causing cell death [6]. The World Health Organization has recognized COL as a critically important antimicrobial agent for human medicine, highlighting its vital role in modern healthcare [7]. However, the recent discovery and global dissemination of the plasmid-mediated mobile COL resistance gene (*mcr-1*) and its variants (*mcr-2* to *mcr-10*) have dramatically compromised COL’s utility. The detection of *mcr*-positive strains across multiple reservoirs in over 50 countries highlights the urgent need for innovative strategies to overcome this resistance mechanism [8–10]. Therefore, innovative measures are urgently needed to prevent the further spread of *mcr-1*-positive bacteria and combat the COL resistance crisis.

Compared with the development of new antibacterial drugs with new targets, which require more investment, longer time, and lower returns, the strategy of antibiotic adjuvants proposes a promising and economically feasible strategy to alleviate the development of bacterial resistance and enhance antibiotic efficacy [11]. Plants produce a remarkable array of secondary metabolites with diverse chemical structures and biological activities, making them ideal sources for such adjuvants. For instance, the combination of baicalin and EDTA can increase the activity of COL against MDR *Salmonella* both *in vitro* and *in vivo* [12]. Glabridin was found to be a potential COL adjuvant to restores sensitivity of COL to *mcr-1*-positive *E. coli* by multiple pharmacological mechanisms [13]. Natural flavonoids (7,8-dihydroxyflavonoids, myricetin, and luteolin) enhance the efficacy of COL by disrupting bacterial iron homeostasis [14]. It is noteworthy that most of the currently reported natural products acting as COL adjuvants are flavonoids and phenolic compounds, while alkaloids are relatively scarce. Therefore, the identification of compounds with enhanced efficacy and multi-target mechanisms remains a critical research priority.

**Figure 1.**
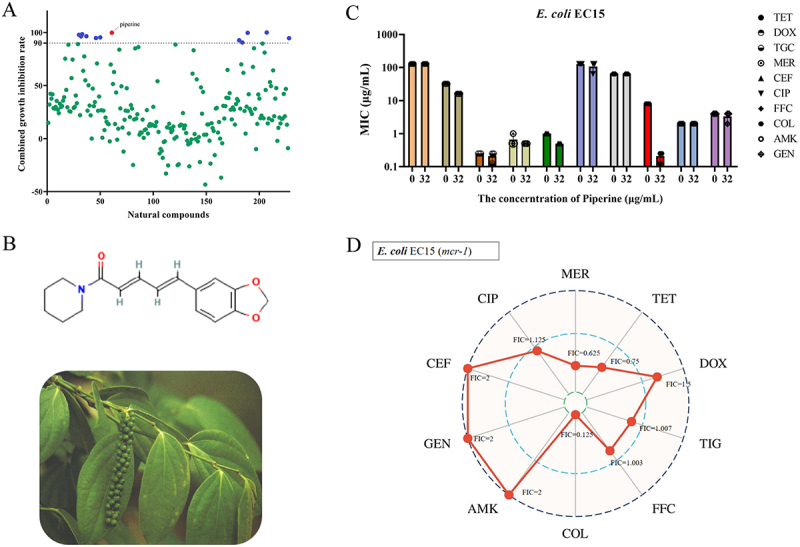
Screening of colistin adjuvants and the synergistic effect between piperine and colistin *in vitro*. (A) Primary screening data on the inhibition of EC15 growth by a combination of 2 μg/mL colistin and 228 molecules from a natural compound library. (B) Chemical structure and source of piperine. (C) MIC of colistin against *E. coli* EC15 in the absence or presence of piperine (32 μg/mL), evaluated across 10 different antibiotic backgrounds. The addition of piperine significantly reduced the MIC of colistin, while no notable effect was observed for other antibiotics. And (D) FICI indices of the combination of piperine (32 μg/mL) and antibiotics of different types against *mcr-1*-positive strains EC15. Note: TET, tetracycline; DOX, doxycycline; TIG, tigecycline; MER, meropenem; CEF, ceftiofur; CIP, ciprofloxacin; FFC, florfenicol; COL, colistin; AMK, amikacin; GEN, gentamicin.

Piperine (PIP), a major bioactive alkaloid from *Piper nigrum* (black pepper) and *Piper longum* (long pepper), represents a particularly promising candidate [15]. This natural compound possesses a unique chemical architecture featuring an aromatic ring, piperidine ring, and aliphatic chain (Figure 1B), which contributes to its diverse pharmacological properties. Previous research has documented piperine’s broad bioactivities, including anti-bacterial, anti-inflammatory and anticancer effects [16–18]. Emerging evidence also suggests its potential in antimicrobial therapy, particularly in enhancing antibiotic activity against Gram-positive bacteria such as *Staphylococcus aureus* and in disrupting biofilm formation [19,20]. However, systematic investigation of piperine’s capacity to reverse COL resistance in Gram-negative pathogens and comprehensive elucidation of its underlying mechanisms remain largely unexplored. In this study, we performed high throughput screening of phytochemical libraries and identified PIP as a potential COL adjuvant. We observed a strong synergistic antimicrobial effect when PIP was combined with COL against MDR Gram-negative bacteria both *in vitro* and *in vivo*. We further elucidated the molecular mechanisms underlying this synergistic bactericidal activity. The discovery of PIP as a new and safe COL adjuvant provides a novel option for combating infections caused by MDR Gram-negative pathogens.

## Materials and methods

### Bacterial strains

A total of 15 non-duplicate COL-reisitant strains, comprising *E. coli* (*n* = 5), *Salmonella* (*n* = 5), *K. pneumoniae* (*n* = 4) and *A. baumannii* (*n* = 1) were preserved from the Pharmacology Laboratory of Henan Agricultural University.    These strains were isolated from pig, chicken, rabbit, and dog fecal samples. All isolates were definitively identified with detailed information provided in Table S1. Additionally, the control strain *E. coli* ATCC 25922 and the engineered strain BL21(DE3) (pET28a-*mcr-1*), were included in the analysis.

### Chemical reagents

Colistin (COL, C4461, ≥19,000 IU/mg) was acquired from Hebei Shengxue Dacheng Bio-pharmaceutical Co., Ltd. (Hebei, China). A total of 228 natural compounds were purchased from MedChemExpress (HY-L0001, purity > 95%, Monmouth Junction, USA). Piperine (PIP, CAS No. 94–62-2, purity > 98 %), was obtained from Macklin (Shanghai, China). Fluorescence probes were sourced from Beyotime Biotechnology Co., Ltd. (Shanghai, China).

### Screening of colistin adjuvants

A total of 228 natural compounds were screened in the presence of 2 μg/mL COL against *E. coli* EC15. Briefly, an overnight bacterial culture was suspended in Mueller-Hinton Broth (MHB) and adjusted to a final concentration of 1 × 10^6^ CFU per well. The bacterial suspension was then distributed into a 96-well flat-bottom plate, followed by the addition of, specified concentrations of COL and the natural products. The microplate was incubated at 37 °C for 18 h, and bacterial growth was quantified by measuring the optical density at 600 nm (OD_600_). Wells containing 2 μg/mL COL (positive control) and 16 μg/mL COL (negative control) were included for comparison. The growth inhibition rate for each test well was calculated as follows: (OD_positive control_ – OD_sample_)/(OD_positive control_ – OD_negative control_) × 100%. The synergistic effect was defined as an inhibition rate of ≥50% [21]. The primary screening was performed in duplicate.

### Antimicrobial susceptibility test

Minimum inhibitory concentration (MIC) was measured using the standard broth microdilution method according to the Clinical and Laboratory Standards Institute (CLSI) guidelines [22]. In brief, COL and PIP underwent two-fold serial dilution in MHB medium. Log-phase bacterial suspensions (1 × 10^6^ CFU/mL) were combined with the diluted drug solutions in a 96-well microplate and incubated at 37 °C for 18 h. The MIC was defined as the minimum concentration of the drug that completely inhibited visible bacterial growth. All experiments were conducted in triplicate.

### Checkerboard assay

The synergistic effect between PIP and COL was assessed using a checkerboard assay. Briefly, COL was serially diluted along the horizontal axis of a 96-well microplate, while PIP was diluted along the vertical axis, creating a two-dimensional concentration matrix. Then, 100 μL of bacterial suspension, standardized to a concentration of 1 × 10^6^ CFU/mL was inoculated into each well. The plate was incubated at 37 °C for 18 h. Each drug combination was performed in at least three independent replicates. The fractional inhibitory concentration index (FICI) was calculated as follows:

$$\mathrm{FICI}\;=\frac{\mathrm{MICab}}{\mathrm{MICa}}+\frac{\mathrm{MICba}}{\mathrm{MICb}}$$

Synergy was defined as a FICI ≤ 0.5 [23]. To determine the stability of the synergistic effect, during seeding into 96‑well plates, exogenous substances including metal ions (Na^+^, K^+^, Mg^2+^, Ca^2+^, Zn^2+^), 10% serum, and 10% DMEM were added to the MHB , while all other steps were kept consistent.

### Growth curves

The test strain was cultured in MHB medium at 37 °C with shaking until OD_600_ reached 0.3. Subsequently, PIP was added to the bacterial suspension to achieve specific final concentrations. The growth kinetics of the bacteria were monitored using a Spark 10 M microplate reader (Tecan). The cultures were maintained at 37 °C with continuous shaking throughout the measurement process, and the absorbance at 600 nm was recorded at hourly intervals for 24 h.

### Time-kill curves

Time-kill experiments were performed to assess the bactericidal efficacy of PIP in combination with COL. Briefly, overnight bacterial cultures were diluted 1:100 in MHB to achieve a starting inoculum of 1 × 10^6^ CFU/mL. These suspensions were then treated with COL alone, PIP alone, or a combination of PIP and COL, for 24 h. Bacterial counts were determined at 0, 2, 4, 8, 12, and 24 h by plating and colony counting. Synergy was defined as a reduction of ≥ 2 log_10_ CFU/mL in the bacterial count by the combination compared to the most effective single agent [24]. All experiments were conducted in triplicate.

### Resistance development study

To assess resistance development, overnight cultures of the tested bacteria were diluted 1:100 in LB broth supplemented with either 0.5 × MIC of COL alone, or a combination of 0.5 × MIC COL and 0.25 × MIC PIP. After 12 h of incubation, the bacterial cultures were diluted 1:100 and transferred into fresh medicated medium for the next passage. The MIC of the passaged cultures was determinedevery two passages over a 24-day period of serial subculturing. All experiments were performed in triplicate to ensure result reliability.

### Live/Dead bacteria staining

Bacterial viability was assessed using a live/dead staining kit to visually distinguish between live and dead cells. Mid-logarithmic phase *E. coli* EC15 was treated with COL (2 μg/mL), PIP (64 μg/mL), or their combination for 4 h at 37 °C. Following the manufacturer’s protocol, live and dead cells were labeled with green and red fluorescence, respectively. The stained cells were then examined under a fluorescence microscope (EVOS M5000, Thermo Fisher Scientific).

### Scanning electron microscopy

Scanning electron microscopy (SEM) was employed to investigate the effects of PIP on bacterial morphology [25]. In brief, bacterial cells in the exponential phase were treated with COL alone or its combination with PIP at 37 °C for 4 h. Next, bacterial cells were collected by centrifugation, washed with PBS, and fixed overnight with glutaraldehyde. Subsequently, the samples were dehydrated through a graded ethanol series, subjected to critical-point drying, and sputter-coated with gold. Finally, the processed samples were imaged using an SEM (JEOL JSM-IT700HR). All SEM imaging and sample processing were conducted by Chengdu Lilai Biotechnology Co., Ltd.

### Mutation preventive concentration assay

Various concentrations of COL alone or in combination with PIP were incorporated into LB agar plates. Subsequently, 100 μL of *K. pneumoniae* KP5 and *E. coli* EC15 (1.0 × 10^10^ CFU/mL) were plated onto the corresponding drug-containing agar plates and incubated at 37 °C. The mutation preventive concentration (MPC) was defined as the lowest drug concentration at which no resistant mutant colonies appeared after 72 h of incubation [26].

### Biofilm inhibition test

The inhibition of biofilm formation was evaluated using the crystal violet staining method [27]. Overnight cultures of the tested bacteria cultures were incubated in TSB broth supplemented with 1% glucose and then diluted 1:100. Subsequently, COL alone, PIP alone or their combination was added to the bacterial cultures, which were then transferred to a 24-well plate. After 48 h of incubation, the biofilms underwent a multi-step treatment protocol involving washing, fixation with methanol, staining with crystal violet, a subsequent wash, decolorization with ice-cold ethanol. Finally, the optical density of the dissolved dye was measured at 595 nm.

### Biofilm eradication assay

The efficacy of PIP in combination with COL against pre-formed mature biofilms was evaluated according to a previously described method with minor modifications [27]. Briefly, mature biofilms were established using COL resistant bacterial strains. After incubation, the supernatant was carefully removed, and the biofilms were gently washed three times with sterile PBS to remove non-adherent planktonic cells. Fresh medium containing COL alone, PIP alone, or their combination was then added to the respective wells, followed by a further 24 h incubation at 37 °C. The remaining biofilms were quantified using the crystal violet staining method as described previously. All experiments were performed in triplicate.

### Confocal laser scanning microscopy

The architecture and viability of *A. baumannii* biofilms were examined using confocal laser scanning microscopy (CLSM) following a published protocol [28]. Briefly, *A. baumannii* PD18 was cultured in TSB medium containing COL, PIP, or their combination in 24–well plates equipped with coverslips. After 48 h of static incubation at 37 °C to allow biofilm formation, the coverslips were gently washed three times with PBS to remove planktonic cells. The adherent biofilms were then stained using the LIVE/DEAD BacLight Bacterial Viability Kit (Thermo Fisher Scientific, China) according to the manufacturer’s instructions. Following staining, excess dye was removed by performing three additional washes with PBS. The prepared samples were finally imaged using a CLSM (LSM800, ZEISS, Jena, Germany).

### Membrane fluidity assays

*E. coli* EC15 cells at an OD_600_ of 0.5 were stained with 10 µM Laurdan, a polarity-sensitive fluorescent probe that partitions into the bacterial cell membrane, and incubated at 37 °C for 10 min in the dark. After staining, the cells were washed three times with PBS buffer and then treated with PIP alone, COL alone, or the combination of PIP and COL at 37 °C for 30 min, with benzyl alcohol serving as the positive control. All treatments were performed on intact bacterial cells without protoplast preparation. Fluorescence intensities were measured using a Spark 10 M microplate reader (Tecan) with an excitation wavelength of 350 nm and emission wavelengths of 460 nm and 500 nm. The Laurdan generalized polarization (GP) value was calculated according to the formula: Laurdan GP = (I_460_ - I_500_) / (I_460_ + I_500_) [13].

### Intracellular protein release

*E. coli* EC15 suspension with an OD_600_ nm value of 0.5 was mixed with COL, PIP and COL+PIP, assemblies in PBS at 37 °C for 2 h. After that, all samples were centrifuged at 5000 rpm for 5 min, and supernatants were collected to measure the total protein content using the BCA kit.

### Swimming motility assay

The effect of PIP on bacterial motility was assessed using 0.3% agar plates containing PIP at concentrations ranging from 0 to 256 µg/mL. A 2 µL aliquot of a bacterial suspension was inoculated onto the center of each plate. After 48 h of incubation at 37 °C, the diameter of the motility halo was measured [29].

#### Biochemical parameters measurement

Biochemical parameters were measured using a fluorescence-based assay. Bacterial samples were processed according to established procedures. An overnight culture of *E. coli* EC15 was washed and resuspended in 5 mM HEPES buffer (pH 7.0) containing 5 mM glucose to an OD_600_ of 0.5. Aliquots (1 mL) of this suspension were treated with COL alone, PIP alone, or their combination, and incubated for 2 h. Subsequently, a fluorescent dye was added and the samples were incubated for another 30 min. Then, 200 μL of each sample was transferred to a white flat-bottomed 96 well plate. Fluorescence intensity or luminescence was measured using a Spark 10 M microplate reader (Tecan).

#### Outer membrane permeability assay

Outer membrane permeability in *E. coli* EC15 was evaluated by the 1-N-phenylnaphthylamine (NPN, 10 μM) uptake assay. The excitation and emission wavelengths were set at 350 nm and 420 nm, respectively [30]. Triton X-100 served as the positive control.

#### Inner membrane permeability

The integrity of the inner membrane was evaluated by propidium iodide (PI, 0.5 mM) uptake. Fluorescence was measured at excitation/emission wavelengths of 535/615 nm [25]. Triton X-100 served as the positive control.

#### Proton motive force assay

Membrane potential and the intracellular pH gradient were assessed. Fluorescence was measured at excitation/emission wavelengths of 622/670 nm for DiSC_3_(5) and 488/535 nm for BCECF-AM [29].

#### EtBr efflux assay

The potential inhibitory effect of PIP and COL on efflux pump activity was evaluated using an ethidium bromide (EtBr, 5 μM) accumulation assay. Carbonyl cyanide 3-chlorophenylhydrazone (CCCP) was used as the positive control. The intracellular accumulation of EtBr was quantified via fluorescence measurement at an excitation wavelength of 530 nm and an emission wavelength of 600 nm [29].

#### NAD^+^/NADH determination

The quantification of NAD^+^and NADH content was performed using an NAD^+^/NADH Assay Kit with WST-8, following cell lysis according to the manufacturer’s instructions.

#### Intracellular ATP

Intracellular ATP levels in *E. coli* EC15 were quantified using an Enhanced ATP Assay Kit (Beyotime, China). After a 2 h exposure to PIP, COL, or their combination, the bacterial cultures were centrifuged. The supernatant, was discarded and the bacterial pellets were lysed with lysozyme. The lysates were then centrifuged again, and the resulting supernatants were collected. Finally, the ATP assay kit (Beyotime, China) was used to detect the concentration of ATP.

#### Total reactive oxygen species and H_2_O_2_ measurement

Intracellular reactive oxygen species levels were measured using 2,7-dichlorodihydrofluorescein diacetate (DCFH-DA, 10 mM) [30]. Fluorescence intensity was measured at excitation and emission wavelengths of 488 nm and 525 nm, respectively. In addition, H_2_O_2_ levels of tested strains were determined using a Hydrogen Peroxide Assay Kit (Beyotime, China). After incubation for one hour, the absorbance of lysis buffer at 560 nm was measured.

### Molecular docking analysis

The 3D structures of MCR-1 (PDB: 5GRR) and PIP were retrieved from the RCSB Protein Data Bank (https://www.rcsb.org/) and PubChem (https://pubchem.ncbi.nlm.nih.gov/), respectively. Molecular docking of the flexible PIP ligand into the rigid MCR-1 receptor was performed using AutoDock Vina 1.1.2 with an exhaustiveness value of 100. The binding affinity was used to evaluate the docking results, where a lower calculated value indicates stronger binding and higher stability of the ligand-receptor complex. Finally, the specific binding interactions and residues involved at the binding site were visualized and analyzed using PyMOL. Based on molecular docking results, the potential binding site of PIP to MCR-1 (Ser284) was mutated to alanine. Briefly, the pET28a-*mcr-1* plasmid was used as a template. Site-directed mutagenesis primers (Table S2) were used to construct the pET28a-*mcr-1* mutant (Ser284Ala). The mutant was confirmed by DNA sequencing and then transformed into *E. coli* DH5α competent cells. The synergistic activity of PIP and COL against the mutant was determined using a checkerboard microdilution assay.

### Western blot

An overnight culture of *E. coli* strain BL21(DE3) harboring the pET28a*-mcr-1* plasmid, which encodes an N-terminal 6×His-tagged protein, was diluted 1:100 in LB broth and incubated at 37 °C for 4 h. The cultures were then induced with 0.4 mmol/L IPTG and simultaneously treated with PIP at concentrations of 0, 16, 32, and 64 µg/mL, followed by another 4 h incubation. Subsequently, the bacterial cells were harvested by centrifugation and lysed by boiling in 2× SDS loading buffer. Total proteins (10 µg per lane) were separated by SDS-PAGE and transferred onto a PVDF membrane. The membrane was blocked and then incubated sequentially with the following primary antibodies: a His-tag mouse monoclonal antibody (Proteintech, Cat. No. 66,005) and a GAPDH mouse monoclonal antibody [GA1R] (Proteintech, Cat. No. 60,004), marker (LABLEAD, Cat. No. P1018). After washing, the membrane was incubated with an HRP-conjugated goat anti-mouse IgG (H+L) secondary antibody (Bioworld, Cat. No. BS12478). The protein bands were finally visualized using a Tanon-5200 chemiluminescence imaging system.

### The impact of piperine on the structure of MCR-1

Ultraviolet absorption spectroscopy was employed to evaluate the binding of PIP to MCR-1. Changes in the UV absorption spectrum of MCR-1 were monitored after incubation with increasing concentrations of PIP [31].

### Safety evaluation

The safety profile of the PIP and COL combination was evaluated through hemolytic and cytotoxicity assays. Hemolytic activity was assessed by incubating mouse red blood cells at 37 °C for 2 h with COL (2 µg/mL), various concentrations of PIP, or their combinations. After incubation, the supernatant was collected, and its optical density was measured at 540 nm. The calculation of the hemolysis rate for each sample was subsequently carried out, and comparisons were drawn against both a positive control (red blood cells in distilled water) and a negative control (red blood cells in physiological saline).

The cytotoxicity of PIP was determined using a CCK-8 kit according to the manufacturer’s instructions. RAW264.7 cells were seeded into 96-well culture plates at a density of 2 × 10^4^ cells per well and incubated overnight at 37 °C. Next day, the cells were treated with various PIP concentrations for 6 h. The Absorbance at 450 nm was measured by the Spapk 10 M microplate reader (Tecan).

### Transcriptome and proteomic analysis

Transcriptome and proteomic analyses of *E. coli* EC15 were performed after exposure to PIP and COL. Overnight bacterial cultures were diluted 1:100 in LB broth and incubated at 37 °C for 4 h to reach the mid-logarithmic growth phase. The cells were then treated with COL (2 μg/mL) alone or in combination with PIP (64 μg/mL) and incubated with shaking at 37 °C for another 4 h. Subsequently, the cells were washed three times with PBS and harvested by centrifugation at 5,000 rpm for 5 min. Finally, the cell pellets were frozen in liquid nitrogen and sent to Majorbio Bio-pharm Technology Co., Ltd (Shanghai, China) for transcriptome and proteomic analysis.

### RT-PCR analysis

*E. coli* EC15 were grown to the early-exponential phase and then treated with COL (2 μg/mL) alone or in combination with PIP (64 μg/mL) for 4 h. Total RNA was extracted from the treated cells, quantified by measuring the A260/A280 ratio, and reverse-transcribed into cDNA using HiScript IV RT SuperMix (Vazyme) according to the manufacturer’s protocol. Quantitative reverse transcription PCR (qRT-PCR) was performed on a 7500 Fast RT-PCR System (Applied Biosystems) using ChamQ Universal SYBR qPCR Master Mix (Vazyme) and gene-specific primers (Table S2). The relative fold changes in gene expression were calculated using the 2^(-ΔΔCt) method, with 16S rRNA serving as the internal reference gene.

### Animal ethics statement

All animal experiments were conducted in strict accordance with the Guidelines for the Use of Laboratory Animals of Henan Agricultural University. The approval number is: HNND2022030905. All animals were treated humanely in compliance with the National Institutes of Health guidelines for the use of experimental animals. This study adhered to the “ARRIVE” guidelines.

### Animal experiments

To evaluate the synergistic effect of PIP and COL on *E. coli in vivo*, a mouse intraperitoneal infection model was used. Pathogen-free BALB/c mice (8 weeks old, weight 22 ± 2) were purchased from Liaoning Changsheng Biotechnology Co., Ltd. (Liaoning, China) and housed at the Animal Research Center of Henan Agricultural University (Zhengzhou, China). All mice were adapted to a constant temperature chamber (22 ± 2 °C) environment for 7 days, with a 12 h light/12 h dark cycle and free access to food and water. A total of 84 mice were used in this study. Forty-eight mice were used in the experiments to planar colony counting and 36 mice were used to histopathological examination. The animals were randomly allocated to experimental groups and processed for blind evaluation. Only the people in charge of data analysis know the grouping. Place the mice population in cages and label the cages sequentially to avoid confusion.

Six-week-old BALB/c mice were randomly allocated into six groups (*n* = 8 per group). Mice were intraperitoneally challenged with an *E. coli* EC15 suspension (1 × 10^8^ CFU/mL). At one hour post-infection, they were administered one of the following treatments via intraperitoneal injection: PBS (control group), COL (5 mg/kg), PIP (5 mg/kg), PIP (10 mg/kg), or a combination of COL and PIP. Survival was monitored every 12 h for 120 h.

In a parallel experiment, six additional groups (*n* = 6) were infected with a sublethal dose of *E. coli* EC15 (1 × 10^5^ CFU/mL) and treated as described above. On day 2 post-infection, the mice were anesthetized with isoflurane and euthanized by cervical dislocation. The liver, kidneys, spleen, and cecum were aseptically collected, weighed, homogenized, and plated for bacterial burden quantification. Serum levels of inflammatory cytokines were quantified using commercial ELISA kits (eBioscience, Affymetrix).

### Statistical analysis

Data were analyzed using SPSS version 21.0 (IBM, Armonk, USA). Graphical representations were generated using GraphPad Prism 10.1.2 (GraphPad Software, USA). T-test, two-way ANOVA test, and Mantel-Cox test were used to perform statistical analysis of the data. Statistical significance was denoted as follows: *p < 0.05, **p < 0.01, ***p < 0.001.

## Results

### Piperine could reverse the resistance of MDR Gram-negative bacteria to colistin without inducing the development of bacterial resistance in vitro

**Figure 2.**
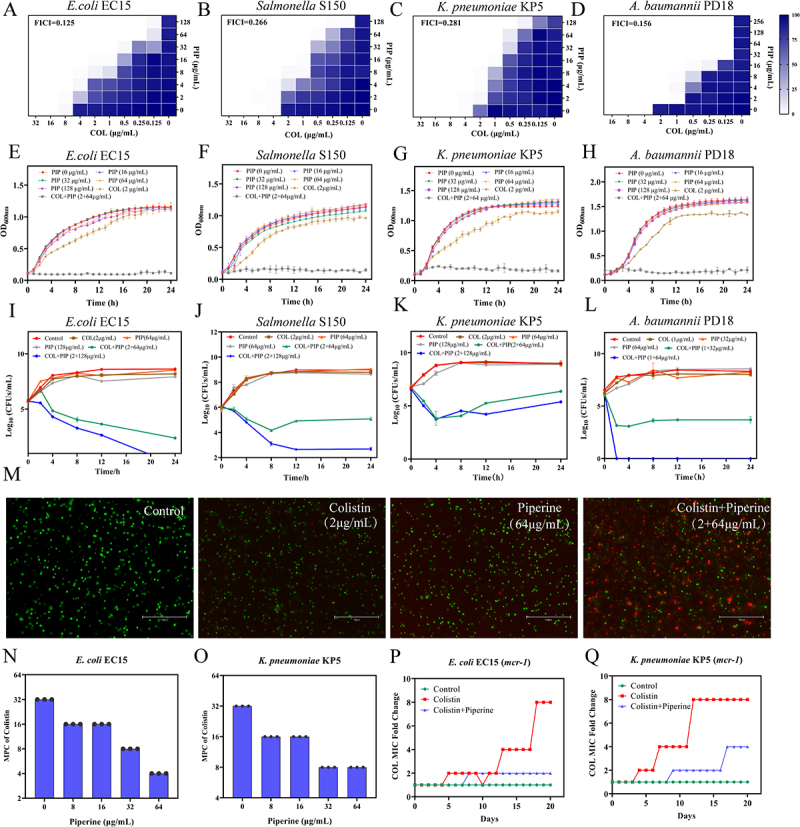
The synergistic effect of piperine in combination with colistin *in vitro*. (A-D) checkerboard analysis showed the combined effect of piperine and colistin against *mcr-1*-positive strains (*E. coli* EC15, *Salmonella* S150, *K. pneumoniae* KP5 and *A. baumannii* PD18). (E-H) growth curves of the four *mcr-1*-positive strains in response to different concentrations of piperine (0 to 128 μg/mL), colistin (2 μg/mL) and colistin (2 μg/mL) in combination with piperine (64 μg/mL). (I-L) Time-killing assays of control, colistin, piperine, and combination treatment against *mcr-1*-positive strains (*E. coli* EC15, *Salmonella* S150, *K. pneumoniae* KP5 and *A. baumannii* PD18, respectively). (M) fluorescence microscope images of colistin-resistant *E. coli* EC15 were treated with piperine (64 µg/mL), colistin (2 µg/mL) the combination or control (PBS), and live bacteria were stained green and dead bacteria were stained red (scale bar = 150 μm). (N, O) MPC values of colistin in the presence of increasing concentrations of piperine against *E. coli* EC15 (*mcr-1*) and *K. pneumoniae* KP5 (*mcr-1*), respectively. Data were obtained from three biological replicates. (P, Q) resistance development assays of *E. coli* EC15 (*mcr-1*) and *K. pneumoniae* KP5 (*mcr-1*) after sequential passages with colistin alone or in combination with piperine, respectively.

Initial screening of 228 monomeric natural compounds revealed that 11 candidates, in combination with COL, exhibited over 90% antibacterial efficacy against *E. coli* EC15 (Table S3). Among these hits, PIP was selected for further investigation as the most promising COL adjuvant due to its potent synergistic activity, established extraction protocols, and low cost (Figure 1A, B). To this end, the synergy between PIP and COL was quantitatively evaluated using both broth microdilution and checkerboard assays. Checkerboard analysis confirmed that PIP (32 µg/mL) synergistically restored COL activity against *E. coli* EC15, with a FICI of 0.125. In contrast, no synergistic effects were observed between PIP and other kinds of antibiotics tested (Figure 1C, D). We expanded our investigation to include COL-resistant strains of *Salmonella*, *K. pneumoniae* and *A. baumannii*. Remarkably, PIP consistently exhibited synergistic interactions with COL across all strains tested, with FICI values ranging from 0.07 to 0.281 (Figure 2A-D; Table 1). Taken together, these findings demonstrate that PIP acts as a potent adjuvant with high specificity and broad-spectrum potential to restore COL activity. We next assessed whether the observed synergy was attributable to antibacterial effects of PIP alone. Growth curve analysis revealed that PIP, at concentrations up to 128 µg/mL, did not significantly inhibit the growth of any tested strains (Figure 2E-H). In contrast, the combination of COL and PIP resulted in pronounced bacterial suppression, which indicates that the synergy is not due to PIP’s intrinsic antibacterial activity.

**Table 1.** MIC values for colistin and FICI values of combination with piperine.

Species	Strains	*mcr-1*	Alone (µg/mL)		Combination(µg/mL)		FICI	Interpretation
			Colistin	Piperine	Colistin	Piperine		
*E. coli*	EC11	+	8	512	1	8	0.141	synergistic
	EC15	+	8	512	1	16	0.156	synergistic
	EC17	+	4	512	0.5	8	0.141	synergistic
	PE77	+	4	512	1	16	0.281	synergistic
	T28R	+	4	512	0.5	32	0.188	synergistic
	BL21(DE3) (pET28a-*mcr-1*)	+	4	512	1	16	0.281	synergistic
*Salmonella*	SK34	+	4	512	1	16	0.281	synergistic
	S150	+	4	512	1	8	0.266	synergistic
	SMG	+	4	512	0.5	8	0.141	synergistic
	SF03	–	8	512	1	32	0.188	synergistic
	TS2a	–	4	512	0.25	4	0.070	synergistic
*K. pneumoniae*	KP12	–	4	512	0.25	16	0.091	synergistic
	KP5	+	4	512	1	16	0.281	synergistic
	KP7	+	32	512	2	32	0.125	synergistic
	KP3	+	4	512	1	8	0.266	synergistic
*A. baumannii*	PD18	–	4	512	0.5	16	0.156	synergistic

Time-kill assays were conducted to determine whether the interaction between PIP and COL was bactericidal. While neither PIP nor COL alone showed significant bactericidal activity, their combination with PIP at 128 µg/mL resulted in substantial reductions in viable bacterial counts across all tested strains. After 24 h of treatment, the combination led to decreases of 8.62, 6.33, 3.58, and 8.02 log_10_CFU/mL relative to COL alone (Figure 2I-L), confirming that the combination of PIP and COL functions as a synergistic bactericidal regimen. To visually corroborate these findings, the bactericidal effect was further examined using LIVE/DEAD BacLight staining in *E. coli* EC15. Viable cells with intact membranes were stained green with SYTO-9, whereas membrane-compromised dead cells were stained red with PI. As shown in Figure 2M, a pronounced increase in red fluorescence was observed in bacterial populations treated with the PIP combination with COL, consistent with the extensive bacterial killing quantified in the time-kill assays.

During prolonged antibiotic exposure, bacterial resistance has intensified through evolution [32]. Consequently, we utilized resistance development and MPC assays to evaluate the capacity of PIP to mitigate the evolution of COL resistance. Following 20 consecutive days of serial passage, we observed that PIP significantly retarded the rise in COL MICs against the passaged strains (Figure 2P, Q). Furthermore, the addition of PIP dose-dependently decreased MPC of COL against the corresponding resistant bacteria, indicating that PIP can effectively impede the emergence and subsequent evolution of COL resistance (Figure 2N, O). Taken together, our research results indicated that PIP, as a potential adjuvant to COL, could reverse COL resistance in Gram-negative bacteria.

### Piperine potentiates colistin to suppress and eradicate biofilms in MDR bacteria

**Figure 3.**
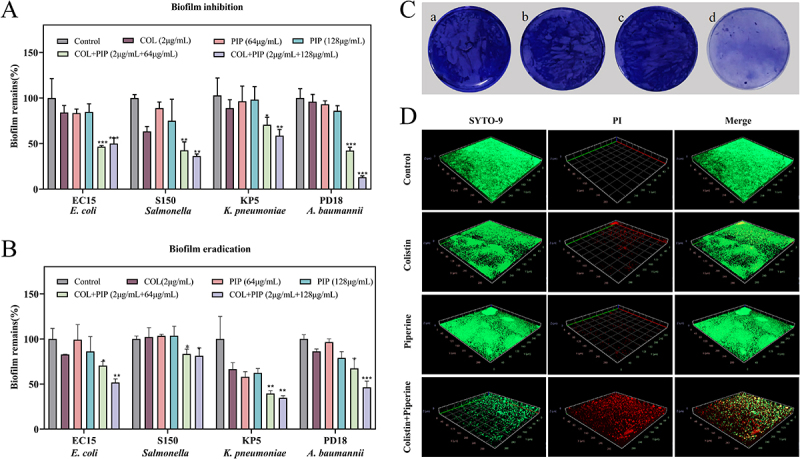
Piperine combination with colistin exhibits biofilm inhibition and eradication activities. Inhibition (A) And eradication (B) Effects of piperine combined with colistin on biofilms of *E. coli* EC15, *Salmonella* 150, *K. pneumoniae* KP5, and *A. baumannii* PD18. (C) Images of crystal violet-stained staining after different treatments (a control, b colistin c, piperine and d, colistin and piperine). (D) 3D CLSM images of biofilms after piperine combined with colistin treatment on biofilm formation of *A. baumannii* PD18 (irradiation intensity: 93.4 mW/cm2, distance: 2 cm). Data are shown as mean ± SD (*n* = 3). *p < 0.05, **p < 0.01, ***p < 0.001.

Biofilms are thought to significantly impede antibiotic efficacy and contribute to treatment failures [33]. Therefore, we evaluated the anti-biofilm potential of the PIP and COL combination using crystal violet staining. As illustrated in Figure 3A, the PIP combination with COL combination significantly inhibited biofilm formation in these strains. Representative staining images of *A. baumannii* PD18 provided visual confirmation of this synergistic inhibitory effect (Figure 3C). Furthermore, the combination regimen demonstrated a remarkable capacity to eradicate pre-formed mature biofilms (Figure 3B), indicating its activity extends beyond prevention to targeting established biofilm structures.

To gain deeper insights into the biofilm architecture and bacterial viability upon treatment, we employed 3D CLSM in conjunction with a live/dead cell viability assay. In this assay, viable cells within the biofilm are stained green with SYTO-9, whereas membrane-compromised dead cells are stained red with PI. As shown in Figure 3D, untreated control biofilms exhibited dense, cohesive structures with predominantly green fluorescence. Monotherapy with either COL or PIP alone induced only minor changes, with a slight increase in red fluorescence. In stark contrast, combination treatment with PIP and COL resulted in a profound disruption of biofilm integrity, characterized by a marked reduction in biofilm density and green fluorescence, coupled with a substantial increase in red fluorescence. These findings visually corroborate that the PIP combined COL synergy not only prevents biofilm formation but also effectively disrupts mature biofilms, leading to widespread bacterial cell death within the biofilm community.

### Piperine potentiates the membrane-disrupting activity of colistin

**Figure 4.**
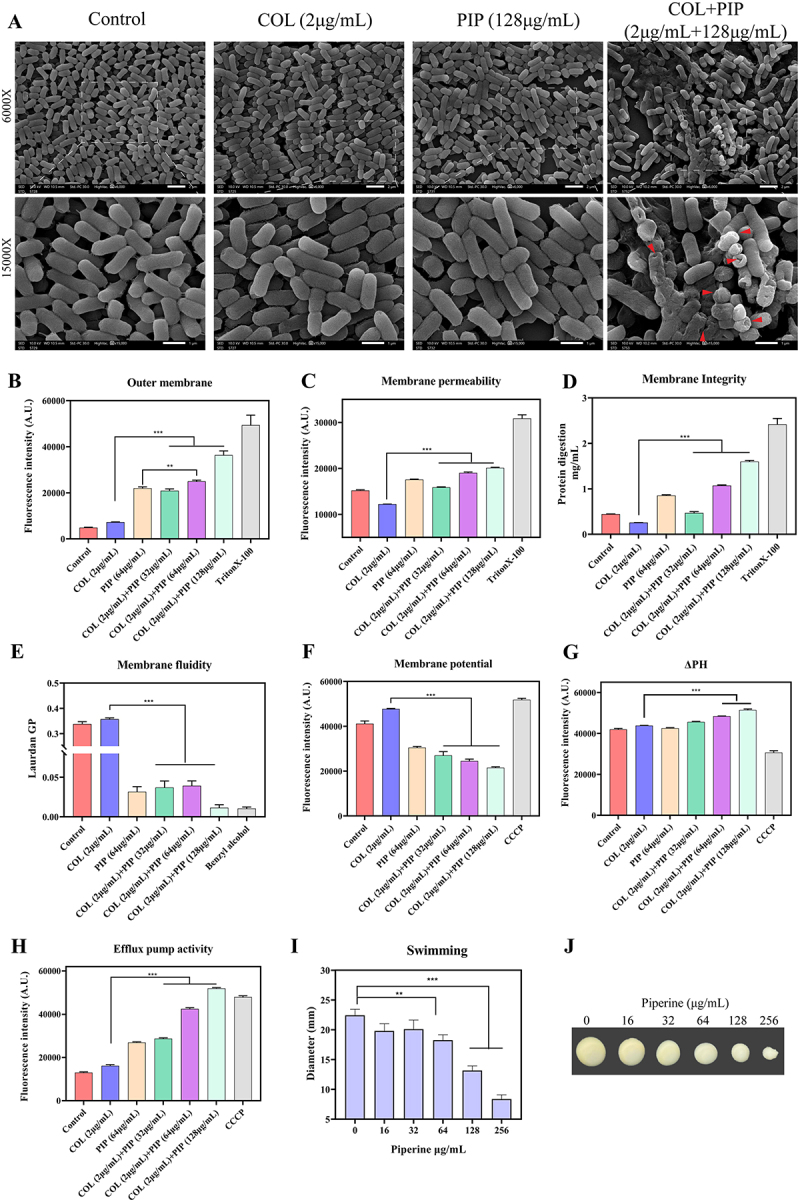
Piperine enhanced colistin damage to the cell membrane. (A) Morphological changes of *E. coli* EC15 treated with colistin (2 μg/mL) or piperine (128 μg/mL) or their combination visualized with SEM. Scar bar, 0.5 µm. Destroyed outer membrane was marked by red arrows. (B, C) The assessment of bacterial membrane integrity by detecting fluorescence values of NPN and PI. TritonX-100 was used as a positive control. (D) Protein leakage from *E. coli* EC15 after various treatments. TritonX-100 was used as a positive control. (E) Membrane fluidity assay. Benzyl alcohol was used as a positive control. (F) Piperine combination colistin causes dissipation of membrane potential. CCCP was used as a positive control. (G) Membrane proton dynamics measured by BCECF-AM fluorescent probe. CCCP was used as a positive control. (H) The efflux pump activity was measured by EtBr, CCCP was used as a positive control. (I, J) Swimming motility of *E. coli* EC15 after piperine treatments. All experiments were performed with biological replicates and presented as mean ± SD (*p < 0.05, **p < 0.01, ***p < 0.001).

The bactericidal action of COL is initiated by its binding to LPS in the outer membrane of Gram-negative bacteria, which increases membrane permeability and leads to the leakage of intracellular contents [34,35]. To determine whether PIP augments this membrane-targeting mechanism, we first examined the morphological changes in *E. coli* EC15 using SEM. As shown in Figure 4A, cells treated with the PIP combination with COL exhibited pronounced morphological damage, including surface depression, collapse, and cell shrinkage (indicated by red arrows), effects that were markedly more severe than those observed with either agent alone. To quantitatively assess membrane damage, we evaluated outer and inner membrane permeability using the fluorescent probes NPN and PI, respectively. The combination of PIP and COL synergistically enhanced the uptake of both probes (Figure 4B, C), demonstrating that PIP significantly potentiates COL-induced disruption of both the outer and inner membranes. BCA protein assay kit analysis revealed increased intracellular protein release following combined treatment with the two agents, which further confirms the membrane-disruptive effect (Figure 4D). In addition, membrane fluidity is a key determinant of bacterial membrane diffusion and transport functions [36]. Our results showed that PIP significantly enhanced bacterial membrane fluidity (Figure 4E), which not only compromises membrane structural integrity but also perturbs intracellular metabolic processes, including the dissipation of PMF [37]. Accordingly, we examined the combined effect of PIP and COL on PMF. The PMF, composed of the electrical potential (Δψ) and the transmembrane proton gradient (ΔpH), is essential for energy generation and cellular homeostasis [38]. Measurement of Δψ and ΔpH using fluorescent probes DiSC_3_(5) and BCECF-AM, respectively. Our results showed that PIP in combination with COL decreased the fluorescence intensity of DiSC_3_(5), indicating that Δψ was disrupted (Figure 4F). In contrast, the introduction of PIP and COL into cells labeled with BCECF-AM led to an increase in fluorescence levels, signifying that PIP combined COL triggered the upregulation of ΔpH in *E. coli* EC15 (Figure 4G). Since bacterial flagellar motility is directly powered by the PMF, we assessed the swimming capability of *E. coli* EC15. As anticipated, PIP treatment resulted in a concentration-dependent inhibition of bacterial motility (Figure 4I, J), confirming the functional consequence of PMF disruption. Given the critical role of PMF in efflux pump activity, we evaluated the function of bacterial efflux pumps in the presence of PIP. The combination of PIP and COL significantly suppressed efflux pump function in *E. coli* EC15, as evidenced by enhanced accumulation of EtBr, an effect comparable to that of the known efflux pump inhibitor CCCP (Figure 4H). These results suggested that PIP enhances the membrane-damaging ability of COL, resulting in collapse of the PMF and efflux pump impairment.

### Piperine synergizes with colistin to enhance the TCA cycle and elicit oxidative damage in bacteria

**Figure 5.**
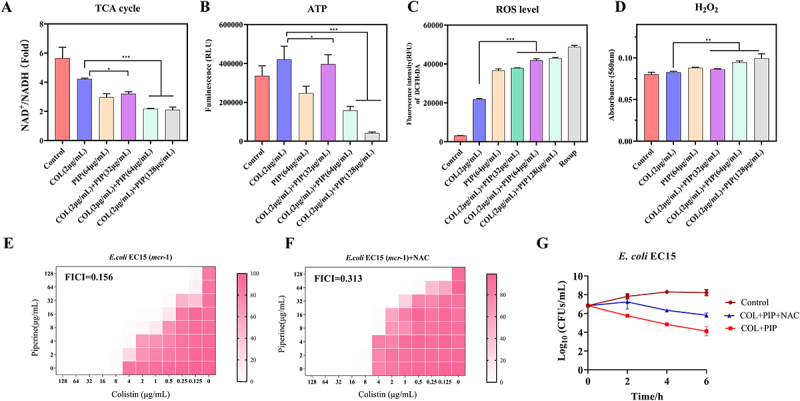
Piperine combined with colistin enhances TCA cycle and ROS accumulation. (A) Effect of different treatment on bacterial intracellular NAD+/NADH levels. (B) *E. coli* EC15 cells treated with piperine and colistin produced less intracellular ATP surveyed by a luciferin-luciferase bioluminescence experiment. (C) A fluorescence probe 2′,7′­dichlorodihydro­fluorescein diacetate (DCFH-DA) was used to monitor the levels of ROS in cells after different treatment. (D) H_2_O_2_ levels at different treatments. (E, F) checkerboard experiments of colistin and piperine against *E. coli* EC15 (*mcr-1*) in the absence or presence of ROS scavengers NAC (10 mM). (G) The synergistic antibacterial effect of colistin and piperine is decreased in the presence of the ROS scavengers NAC (10 mM). All data are presented as mean ± SD, and the significances were determined by T-test and non-parametric oneway ANOVA (*p < 0.05, **p < 0.01, ***p < 0.001).

In addition, we evaluated the intracellular ATP level of *E. coli* EC15, as ATP production is closely associated with membrane potential, which in turn is linked to the PMF generated by the electron transport chain [39]. Our results showed that the combination of PIP and COL significantly reduced intracellular ATP levels (Figure 5B), consistent with PMF dissipation, indicating a critical disruption in bacterial energy metabolism. The cofactors NAD^+^ and NADH play key roles in driving various biochemical reactions, and their ratio serves as a major indicator of bacterial metabolic status [40]. To further confirm whether the combination of PIP and COL accelerates the TCA cycle, we measured the NAD^+^ /NADH ratio. Our observations demonstrated that the addition of PIP led to a significant decrease in these ratios (Figure 5A), suggesting enhanced TCA cycle activity in *E. coli* EC15. Typically, accelerated TCA cycle is accompanied by increased bacterial respiration and ROS production [41]. Endogenous ROS play a critical role in the bactericidal activity of antibiotics; their accumulation disrupts bacterial membrane homeostasis, causes damage to proteins, nucleic acids, and membrane structures, and ultimately induces cell death [42]. Herein, we measured the ROS levels in *E. coli* EC15 following combined treatment and found that ROS levels surged in a concentration-dependent manner (Figure 5C). ROS, which mainly consist of superoxide anions (O_2_•^−^), hydrogen peroxide (H_2_O_2_), and hydroxyl radicals (•OH), play a pivotal role in antibiotic-mediated bacterial killing [43]. Interestingly, we also observed a significant increase in H_2_O_2_ generation following the combined treatment (Figure 5D). To confirm the functional role of ROS in the observed synergy, we introduced N-acetyl-L-cysteine (NAC), a potent ROS scavenger, into the assay systems. The addition of NAC markedly attenuated the synergistic enhancement of COL activity by PIP in both checkerboard and time-kill assays (Figure 5E–G), demonstrating that oxidative damage is a crucial mechanism of action.

### Piperine potentiates colistin by inhibiting the MCR-1 protein and down-regulating its expression

**Figure 6.**
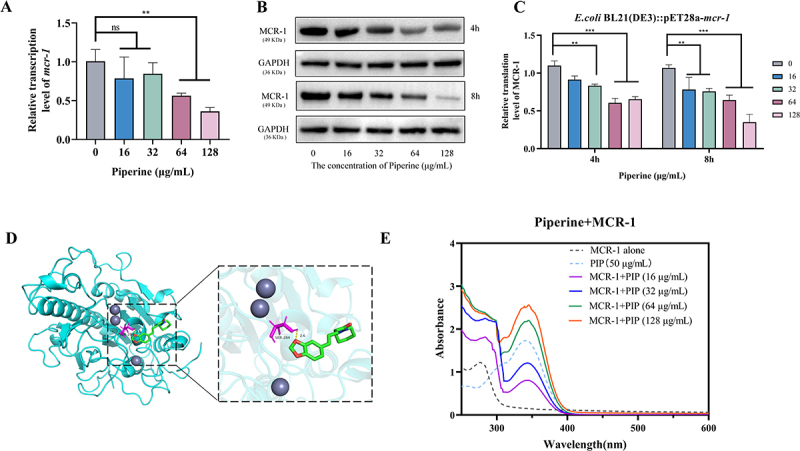
The interaction between piperine and MCR-1. (A) decrease in the relative transcription level of *mcr-1* in EC15 treated with piperine. (B, C) decrease in the relative translation level of *mcr-1* in *E. coli* strain BL21(DE3):pET28a-*mcr-1* treated with piperine. GAPDH is the loading control. (D) the interaction between piperine and MCR-1 was simulated by molecular docking. (E) interaction of piperine and MCR-1 protein in ultraviolet absorption spectrum. Data are presented as the mean ± SD. *p < 0.05, **p < 0.01, ***p < 0.001.

To elucidate the molecular mechanism by which PIP overcomes plasmid-mediated COL resistance, we investigated its effect on the expression and function of the MCR-1 protein. RT-qPCR analysis revealed that treatment with PIP significantly downregulated the transcription of the *mcr-1* gene in a concentration-dependent manner in *E. coli* EC15 (Figure 6A). Consistent with this finding, western blot analysis demonstrated a corresponding dose-dependent reduction in MCR-1 protein levels in PIP treated *E. coli* BL21(DE3):pET28a-*mcr-1* (Figure 6B, C), indicating that PIP suppresses both the synthesis and the steady-state abundance of this key resistance determinant. We next employed molecular docking to explore potential direct interactions between PIP and the MCR-1 protein. The simulation revealed that PIP binds stably within a surface groove of MCR-1, forming a critical hydrogen bond with the Ser284 residue (Figure 6D). To further validate the molecular docking results, the candidate residue Ser284 was mutated to alanine (S284A). The synergistic activity of PIP and COL against the mutants was then evaluated by the checkerboard microdilution method. As expected, the synergistic effect was significantly weaker than that against the wild-type strain (Table S4). This specific interaction suggests that PIP may act as a potential inhibitor by occupying a functionally important site. Furthermore, ultraviolet absorption spectroscopy showed that PIP binding induced notable changes in the protein’s spectral profile (Figure 6E), providing direct biophysical evidence that PIP binding alters the tertiary structure of MCR-1. These results suggested that PIP may restore bacterial sensitivity to COL by inhibiting the expression and function of MCR-1

### Integrated transcriptomic and proteomic analysis reveals multi-faceted mechanisms of synergy

**Figure 7.**
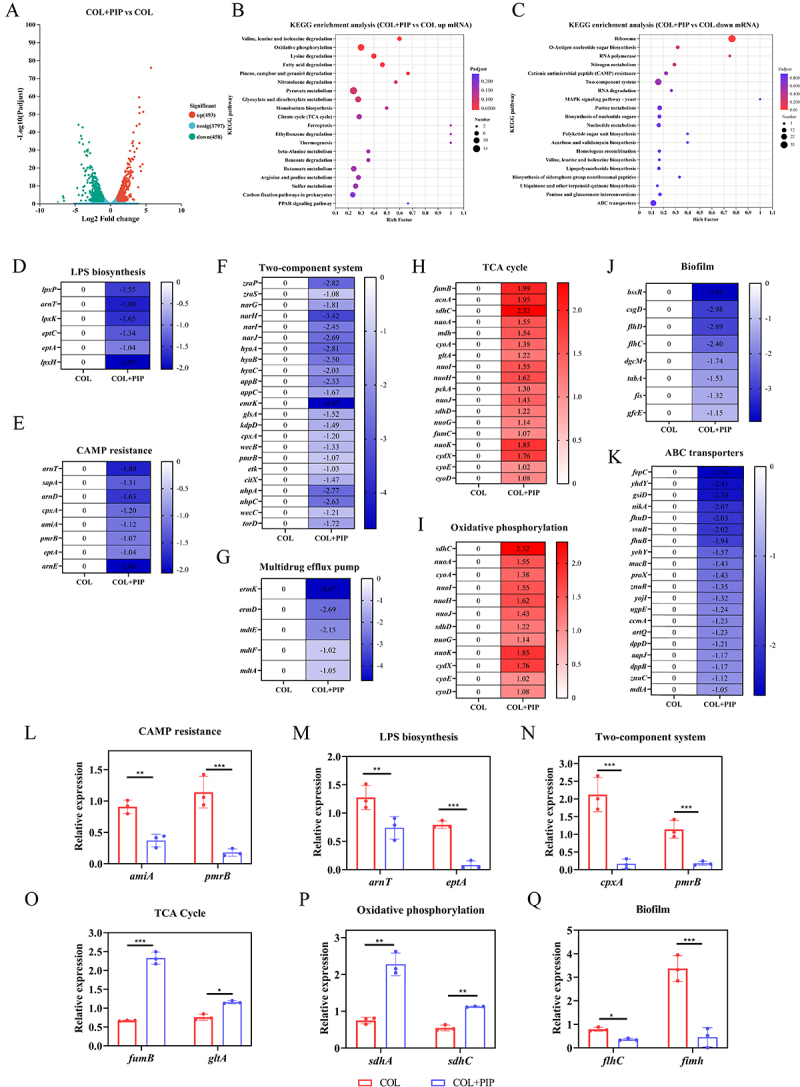
Transcriptomic assessment of *E. coli* EC15 with piperine plus colistin treatment. (A) Volcano plot showing the numbers of upregulated (493) or downregulated (458) DEGs. Then, KEGG enrichment analyses were performed (B) For upregulated pathways and (C) For downregulated pathways. The related pathways of the selected DEGs were enriched, including the downregulated pathways of LPS biosynthesis (D), two-component system (F), Ribosome (G), CAMP resistance (H), biofilm (I), and ABC transporters (J) As well as the upregulated pathways the TCA cycle (E) and oxidative phosphorylation (K). The relative expression levels of representative genes of CAMP resistance (L), LPS biosynthesis (M), two-component system (N), TCA cycle (O), oxidative phosphorylation (P), and biofilm (Q) in *E. coli* treated with 2 μg/mL colistin or 64 μg/mL naringenin plus 1 μg/mL colistin. Data are presented as the mean ± SD. *p < 0.05, **p < 0.01, ***p < 0.001.

To gain a systems-level understanding of the synergistic mechanism between COL and PIP, we performed integrated transcriptomic and proteomic profiling of *E. coli* EC15 after 4 h of exposure to COL alone or in combination with PIP. Transcriptomic analysis revealed 951 differentially expressed genes (DEGs) in the combination group compared to COL monotherapy, comprising 493 upregulated and 458 downregulated genes (Figure 7A). To decipher the functional implications of the transcriptional changes, we conducted KEGG and GO enrichment analyses (Figure S1, Figure 7 B, C). The results demonstrated a systematic downregulation of pathways critical for bacterial defense and homeostasis, including cell envelope biogenesis (LPS biosynthesis; Figure 7D), antibiotic resistance mechanisms (CAMP resistance and two-component systems; Figure 7E, F), multi-drug efflux pump (Figure 7G), biofilm-related pathways (Figure 7J) and ABC transporters (Figure 7K). RT-qPCR validation confirmed these findings at the gene expression level, specifically documenting the downregulation of key resistance genes including CAMP resistance-related *amiA* and *pmrB* (Figure 7L), LPS biosynthesis genes *arnT* and *eptA* (Figure 7M), two-component system genes *cpxA* and *pmrB* (Figure 7N), as well as critical biofilm-associated genes *flhC* (involved in flagellar assembly) and *fimH* (essential for type I fimbria adhesion) (Figure 7Q). This comprehensive suppression of multiple defense mechanisms represents a crucial factor in re-sensitizing bacteria to COL. Concurrently, the combination treatment induced significant metabolic reprogramming, evidenced by the upregulation of the TCA cycle and oxidative phosphorylation pathways (Figure 7H, I). This disrupted metabolic flux was further validated through RT-qPCR analysis of central metabolic genes, including *fumB* and *gltA* in the TCA cycle, as well as *sdhA* and *sdhC* which promote oxidative phosphorylation (Figure 7O, P). The resulting hyperactive metabolic state is closely linked to the ROS-mediated bacterial damage observed in our study, thereby explaining the potent bactericidal synergy.

**Figure 8.**
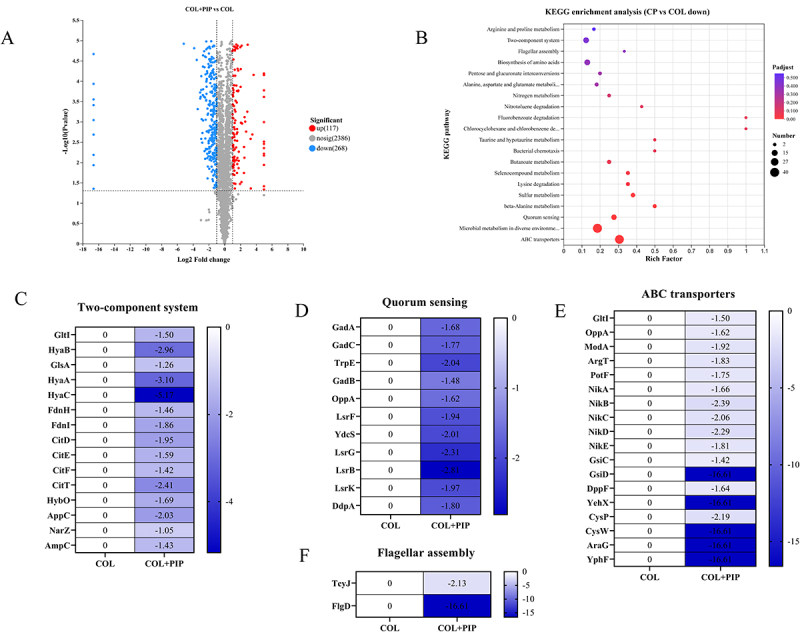
Proteomic analysis of piperine and colistin treatment on *E. coli* EC15. (A) a volcano plot from the proteomic analysis. (B) KEGG enrichment analysis. Pathway schematics illustrating the downregulation of differential proteins involved in the two-component system (C), quorum sensing (D), ABC transporters (E), and flagellar assembly (F) upon colistin and piperine treatment.

Our proteomic analysis demonstrated that the COL and PIP combination treatment profoundly altered the bacterial proteome, resulting in 268 downregulated proteins (Figure 8A). Notably, functional enrichment analysis highlighted that the most significantly suppressed proteins were associated with several key systems: ABC transporters (GsiD, CysW, AraG, YphF), two-component systems (HyaC), quorum sensing, and flagellar assembly (FlgD) (Figure 8B-F). This functional suppression at the protein level is consistent with our transcriptomic findings, revealing a coordinated downregulation of these critical pathways. The concerted downregulation of these specific targets points to a synergistic mechanism that simultaneously attenuates multidrug efflux capacity, disrupts signal transduction and cooperative behaviors, and inhibits bacterial motility, thereby crippling multiple defense essential for bacterial survival.

### Safety and stability evaluation of colistin and piperine combination

The safety of drugs is essential for clinical use. Therefore, we conducted safety experiments to evaluate the toxicity of PIP and COL combination, encompassing hemolytic activity on mammalian red blood cells and cytotoxicity. The results showed that PIP demonstrated low hemolysis rates (about 1.5%) throughout doses of 2 to 128 μg/mL (Figure S2 A). Moreover, the combination of COL (2 μg/mL) and PIP at these concentrations does not cause hemolysis of red blood cells (Figure S2 B). Additionally, PIP exhibited mild cytotoxicity at concentrations below 128 μg/mL (Figure S2 C). Our preliminary research results denote that PIP with a concentration below 128 μg/mL exhibits minimal hemolytic and cytotoxic properties. Nonetheless, more comprehensive research is required to understand the safety of PIP *in vivo.*

The *in vivo* environment contains high concentrations of cations, serum proteins, and complex nutrients, which may interfere with the target binding of COL and alter its efficacy. Therefore, it is necessary to evaluate whether the synergistic activity of the PIP‑COL combination remains stable under simulated physiological conditions. We next evaluated the stability of this combination in the presence of serum, DMEM, or different salt ions using *E. coli* EC15. PIP retained its synergistic activity with COL in the presence of 10% serum or DMEM (Table S5). The presence or absence of monovalent cations (Na^+^, K^+^) did not significantly affect the synergistic activity. EDTA enhanced their synergistic activity, whereas Mg^2+^ and Ca^2+^ moderately reduced the synergy (Figure S3 and Table S5). Collectively, these results demonstrate that the PIP‑COL combination maintains robust synergistic activity under physiologically relevant conditions, supporting its favorable safety and stability profile.

### Piperine potentiates the in vivo efficacy of colistin in a murine infection model

**Figure 9.**
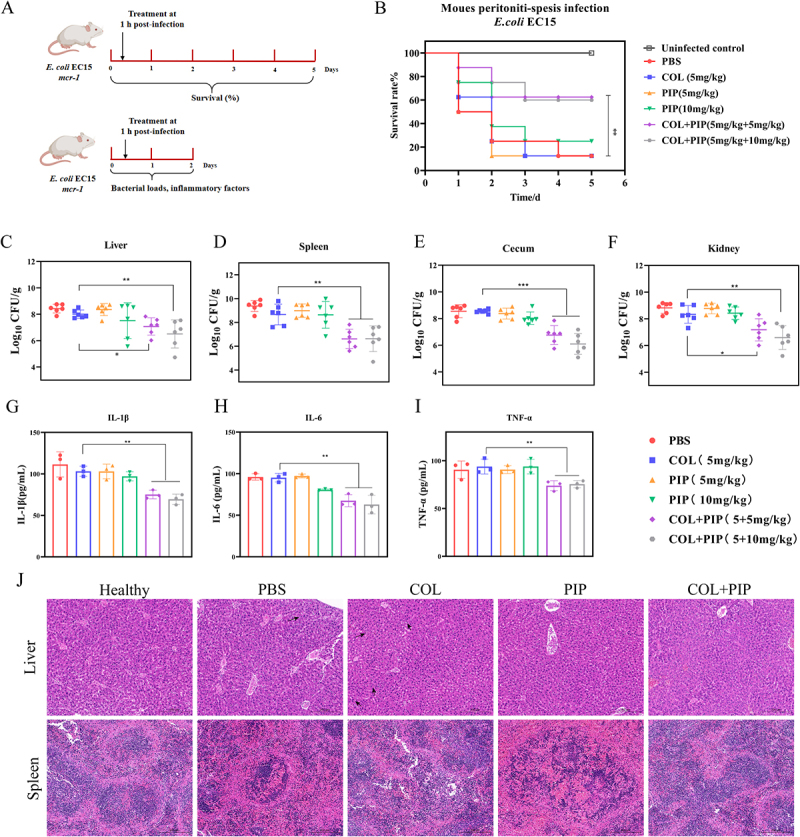
Piperine combined with colistin effectively prevented mice from *E. coli* infection. (A) the experimental scheme of synergy analysis in *E. coli* EC15-infected mice. (B) survival rates of infected mice (*n* = 8 per group) treated by colistin (5 mg/kg), piperine (5, 10 mg/kg), or their combination (5 + 5 or 5 + 10 mg/kg). The mice were infected by *E. coli* EC15 (*mcr-1*). (C-F) at 72 h postinfection with 1 × 10^7^ CFU/g, the bacterial CFU of the liver, spleen, kidney and cecum were calculated. (G-I) serum levels of interleukin IL − 6, TNF-α and IL-1β measured via enzyme-linked immunosorbent assay (ELISA; *n* = 3). (J) the pathological sections of the main organs of mice after different treatments. The scale bar is 200 μm. The results are displayed as the mean ± SD of three biological replicates. *p < 0.05, **p < 0.01, ***p < 0.001.

Based on the demonstrated *in vitro* synergy between PIP and COL against resistant Gram-negative pathogens, we established a murine abdominal infection model to evaluate its therapeutic efficacy *in vivo* (Figure 9A). The PIP combination with COL significantly enhanced host survival, increasing the rate from 12.5% with COL monotherapy (5 mg/kg) to 62.5% (Figure 9B). This protective effect was associated with a substantial reduction in bacterial loads in the spleen, kidney, liver, and cecum compared to the COL alone group (*p* < 0.05; Figure 9C-F). Histopathological examination further confirmed the therapeutic benefit, showing markedly alleviated tissue damage in the combination group as evidenced by hepatocyte inflammatory cell infiltration and splenic damage (Figure 9J). Notably, the combination therapy also produced a significant immunomodulatory effect, substantially attenuating the production of pro-inflammatory cytokines (IL-6, IL-1β, and TNF-α) compared to untreated controls or either agent alone (Figure 9G-I). These collective findings provide compelling evidence that PIP synergizes with COL *in vivo* to enhance bacterial clearance, improve survival outcomes, and modulate detrimental inflammatory responses.

## Discussion

The escalating crisis of antimicrobial resistance among Gram-negative pathogens necessitates innovative strategies to preserve the efficacy of last-resort antibiotics [44]. The development of novel antibiotic adjuvants provides new strategies to address the limitations of existing antibiotics and meet clinical demands [11]. Our previous studies have reported that the combination of COL and adjuvants can effectively extend the therapeutic lifespan of COL and rescue its bactericidal activity against COL-resistant pathogens [12,45,46]. This study reveals that PIP acts synergistically with COL to eradicate bacteria. The remarkable synergy observed between PIP and COL against clinically relevant pathogens including *E. coli*, *K. pneumoniae*, *Salmonella*, and *A. baumannii* (FICI values of 0.07–0.281) underscores the therapeutic potential of this combination. This synergistic effect was validated in both *in vitro* and *in vivo*. The significant nephrotoxicity and neurotoxicity of COL in mammalian cells severely limit its clinical use [47]. Fortunately, PIP is non-hemolytic and shows no significant cytotoxicity toward mammalian cells, thus the application of PIP efficaciously reduced the treatment dosage of COL, which further reduced the risk of side effect.

Biofilm formation constitutes a key mechanism of antimicrobial tolerance, where extracellular polymeric substances provide physical protection and enhance resistance [48]. The demonstrated efficacy of the combination of PIP and COL against both planktonic and biofilm-embedded Gram-negative bacteria thus significantly boosts its clinical relevance. Our multi-omics analysis reveals that this potent anti-biofilm activity operates through a multi-layered mechanism. Phenotypically, the combination effectively inhibits biofilm formation and eradicates mature structures. Molecularly, this is driven by the coordinated suppression of key biofilm machineries: transcriptomic and RT-PCR data showed downregulation of biofilm critical adhesion genes *flhC* and *fimH* upon drug treatment (Figure 7Q). Proteomic data also showed downregulation of quorum sensing systems, and arginine metabolism. This concurrent disruption at the functional, transcriptional, and protein levels undermines both the structural integrity and cell-to-cell communication essential for biofilm development. By simultaneously targeting a key resistance mechanism and a hotspot for its evolution, the combination of PIP and COL presents a robust strategy against persistent infections.

Our mechanistic studies demonstrate that PIP enhances COL-induced membrane damage through multiple pathways of synergistic interaction, which represents a crucial underlying mechanism. Piperine has been reported to increase cell membrane permeability in various bacterial and fungal species [49,50]. In light of the observation that exogenous addition of divalent cations (Ca^2+^, Mg^2+^) or exogenous LPS attenuated the synergistic activity of PIP and COL (Figure S3, Table S5), while EDTA enhanced their synergistic activity against *E. coli* EC15 (Figure S4), we suspected that the synergistic mechanisms may involve disruption of the bacterial outer membrane [41,51]. This membrane-destabilizing possibility was further supported by direct morphological and permeability assays. SEM examination revealed that, compared to COL or PIP alone, the combination induced pronounced cell surface depression, collapse, and shrinkage (Figure 4A), indicating severe structural damage. Fluorescence assays showed that the combination synergistically enhanced both outer and inner membrane permeability, as evidenced by increased uptake of NPN and PI probes (Figure 4B,C), accompanied by elevated leakage of intracellular proteins (Figure 4D). Additionally, PIP significantly increased bacterial membrane fluidity (Figure 4E), a property critical for membrane integrity and transport functions [36]. Enhanced fluidity not only compromises the barrier function of the membrane but may also perturb downstream processes such as PMF and efflux pump activity. These observations are further supported by our multi-omics findings. The significant downregulation of LPS modification genes (*arnT* and *eptA*) suggests that PIP may not only interfere with existing LPS structures but also suppress the bacterial capacity to remodel LPS, thereby compromising resistance to COL. Concurrently, transcriptomic analysis revealed downregulation of two-component system genes (*cpxA* and *pmrB*), which was further confirmed by proteomic data showing suppression of the corresponding proteins. This coordinated suppression at both transcriptional and translational levels likely disrupts the signaling pathways that bacteria use to sense and respond to membrane damage, thereby exacerbating the disruption of the initial membrane structure.

Loss of membrane integrity disrupts transmembrane potential, impairs ion homeostasis, and further exacerbates the imbalance in energy metabolism [52]. The combination of PIP and COL disrupted the PMF homeostasis in *E. coli*, which serves as the essential energy source for a wide range of cellular processes. This PMF collapse has several major effects. It directly inhibits PMF-dependent multidrug efflux pumps, as evidenced by transcriptomic data showing downregulation of key efflux pump genes (*mdtE*, *emrD*, *ermK*) and proteomic analysis revealing suppression of ABC transporters. The disruption of PMF impairs ATP production, creating a severe energy shortage that impairs many cellular functions [53]. This energy crisis is worsened by abnormal metabolic changes. Integrated multi-omics analyses revealed upregulation of the TCA cycle and oxidative phosphorylation pathways, evidenced by increased expression of central metabolic genes including *fumB* and *gltA* in the TCA cycle, as well as *sdhA* and *sdhC* in oxidative phosphorylation (Figure 7O, P). This seemingly contradictory observation may be attributed to multiple factors. Firstly, PIP-induced damage to the cell membrane led to ATP leakage, further exacerbating the energy deficit since active membrane repair is an energy-dependent process. Secondly, PMF was a driver of ATP synthesis, and its collapse affected ATP synthesis [13]. This hyperactive metabolic state, occurring in the context of impaired energy utilization, creates a severe metabolic imbalance that ultimately leads to substantial ROS explosive accumulation [43]. The essential role of oxidative stress in the bactericidal synergy was unequivocally confirmed by the complete abolition of synergistic activity upon addition of the ROS scavenger NAC. This metabolic dysregulation, initiated by membrane damage and PMF collapse, represents a self-amplifying cycle wherein energy impairment leads to metabolic imbalance, which in turn generates lethal oxidative stress, ultimately resulting in bacterial cell death.

A particularly significant aspect of our findings is PIP’s comprehensive strategy against *mcr-1*-mediated resistance, which operates through dual mechanisms to both counteract existing resistance and prevent its further evolution. The dual inhibition of MCR-1 simultaneously suppressing *mcr-1* transcription and translation while directly binding to the MCR-1 protein via hydrogen bonds with the Ser284 residue represents a sophisticated approach to overcoming plasmid-mediated resistance. The demonstration that PIP significantly delays the development of elevated COL resistance in serial passage experiments using *mcr-1*-harboring strains provides compelling evidence for its potential to extend the clinical lifespan of COL. This prevention effect likely stems from PIP’s multi-target action, which increases the genetic fitness cost required for resistance acquisition, thereby creating a higher evolutionary barrier for resistance development.

The therapeutic relevance of our findings is substantiated by robust *in vivo* results demonstrating that the PIP combination with COL significantly improved survival rates, reduced bacterial loads in major organs, and attenuated pro-inflammatory cytokine production in a mice infection model. While formulation challenges exist due to PIP’s poor aqueous solubility, recent advances in drug delivery technologies, including nanoparticle-based systems and lipid formulations, offer promising solutions to enhance its bioavailability and pharmacokinetic properties [54,55].

## Conclusion

**Figure 10.**
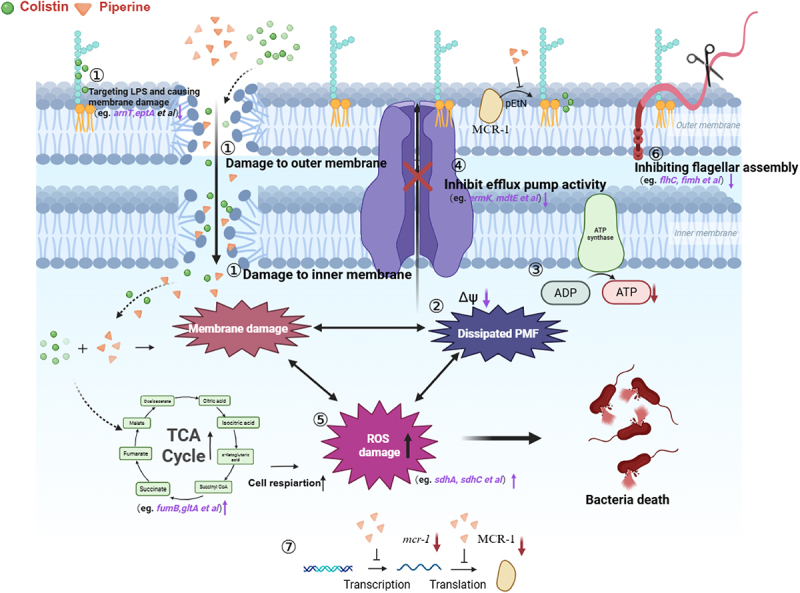
Scheme summarizing the proposed mechanisms that piperine enhancing the antibacterial effect of colistin. (1) Piperine could enhance the ability of colistin to damage by targeting LPS and chelating cations, which punch holes in both the outer membrane and inner membrane. (2) Piperine combined with colistin can dissipate PMF in *E. coli*. (3) Combination treatment disrupted the energy metabolism homeostasis within the bacteria and (4) Inhibited the function of the efflux pump. (5) Accumulation of intracellular ROS by accelerating the tricarboxylic acid cycle and membrane disruption. (6) Combination treatment results in the downregulation of flagellar assembly-related genes and defective of flagellum. (7) Piperine could be developed as the a MCR-1 inhibitor, which contribute to reverse the colistin resistance of *mcr-1*-harboring *E. coli* strains. Multiple mechanisms of action work together to enhance the antibacterial activity of piperine against MDR pathogens.

This study identified that PIP, a natural alkaloid, drastically potentiated COL activity against MDR Gram-negative pathogens and is less prone to the emergence of drug resistance. The mechanism study has shown promising results in reversing COL resistance through multiple mechanisms (Figure10), involving simultaneous membrane disruption, PMF dissipation, efflux pump inhibition, metabolic interference, accumulation of ROS and direct targeting of MCR-1. Notably, PIP exhibits strong safety and significantly enhances the *in vivo* efficacy of COL. Our findings offer a viable strategy to facilitate and accelerate the clinical application of antibacterial synergists.

## Supplementary Material

Clean Copy of Supplementary Material - QVIR-2025-1107.R2.docx

## Data Availability

RNA-Seq analysis data have been deposited in the National Center for Biotechnology Information (NCBI) Sequence Read Archive (SRA) database (PRJNA1225638). The proteomics data have been deposited to the ProteomeXchange Consortium via the PRIDE partner repository with the dataset identifier PXD072876. The raw data that support the findings of this study are openly available in figshare at https://doi.org/10.6084/m9.figshare.30885389 [56].
