# Supplementary material for: Piperine reverses colistin resistance in multidrug resistant Gram-negative pathogens by membrane disruption and ROS damage
Source: Virulence. 2026 Jun 30;17(1):2691333. doi: 10.1080/21505594.2026.2691333 (PMC13336263; doi:10.1080/21505594.2026.2691333)
Supplement: Clean Copy of Supplementary Material - QVIR-2025-1107.R2.docx [file KVIR_A_2691333_SM5220.docx]

**Piperine reverse colistin resistance in multidrug resistant Gram-negative pathogens by membrane disruption and ROS damage**

**Supplementary materials**

**Supplementary Tables**

**Table S1 The information and resistance spectrum of the strains used in this study**

| Species | strain | *mcr-1* | Antibiotic resistance | | | | | | | | | | Source |
| --- | --- | --- | --- | --- | --- | --- | --- | --- | --- | --- | --- | --- | --- |
|  |  |  | Colistin | Meropenem | Tetracycline | Doxycycline | Tigecycline | Florfenicol | Amikacin | Gentamicin | Ceftiofur | Ciprofloxacin |  |
| *E. coli* | EC15 | + |  |  |  |  |  |  |  |  |  |  | Swine |
|  | EC17 | + |  |  |  |  |  |  |  |  |  |  | Swine |
|  | EC11 | + |  |  |  |  |  |  |  |  |  |  | Swine |
|  | PE77 | + |  |  |  |  |  |  |  |  |  |  | Swine |
|  | T28R | + |  |  |  |  |  |  |  |  |  |  | Dog |
| *Salmonella* | SK34 | + |  |  |  |  |  |  |  |  |  |  | Swine |
|  | TS2a | - |  |  |  |  |  |  |  |  |  |  | Chicken |
|  | SMG | + |  |  |  |  |  |  |  |  |  |  | Chicken |
|  | S150 | + |  |  |  |  |  |  |  |  |  |  | Chicken |
|  | SF03 | + |  |  |  |  |  |  |  |  |  |  | Swine |
| *K. pneumoniae* | KP5 | + |  |  |  |  |  |  |  |  |  |  | Rabbit |
|  | KP7 | + |  |  |  |  |  |  |  |  |  |  | Rabbit |
|  | KP12 | + |  |  |  |  |  |  |  |  |  |  | Rabbit |
|  | KP3 | + |  |  |  |  |  |  |  |  |  |  | Rabbit |
| *A. baumannii* | PD18 | - |  |  |  |  |  |  |  |  |  |  | Chicken |

Note: deep red represents bacteria resistant to this drug, while blue represents sensitivity.

**Table S2 Natural Compounds Exhibiting High Synergy (≥90% Inhibition) with Colistin Against *E. coli* EC15.**

|  | Phytochemicals | CAS number | Structure | Combined growth inhibition rate (%) |
| --- | --- | --- | --- | --- |
| 1 | Theophylline | 58-55-9 | 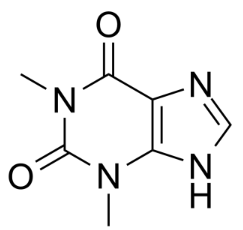 | 92.63 |
| 2 | 4-O-Methyl honokiol | 68592-15-4 | 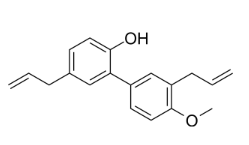 | 90.57 |
| 3 | Chelerythrine | 34316-15-9 | 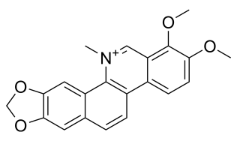 | 96.65 |
| 4 | Pinosylvin | 22139-77-1 | 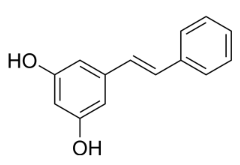 | 97.77 |
| 5 | piperine | 94-62-2 | 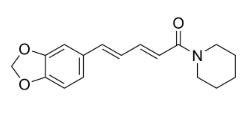 | 99.78 |
| 6 | 8-Gingerol | 23513-08-8 | 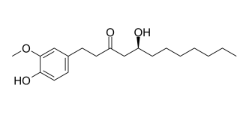 | 95.33 |
| 7 | Karacoline | 39089-30-0 | 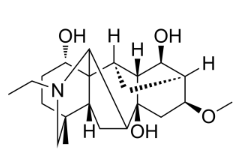 | 98.32 |
| 8 | Ellipticine | 519-23-3 | 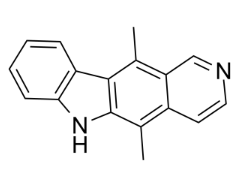 | 96.46 |
| 9 | Harmane | 486-84-0 | 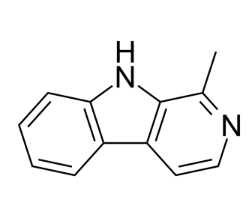 | 94.79 |
| 10 | (-)-Alkannin | 517-88-4 | 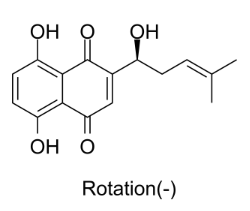 | 96.27 |
| 11 | Coronaridine | 467-77-6 | 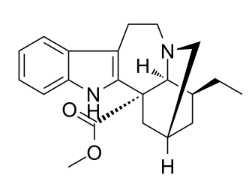 | 94.61 |

**Table S3 Primers used in this study**

| **Primer** | **Primer sequence(5′→3’)** | **References** |
| --- | --- | --- |
| *mcr-1*-qF | ACACTTATGGCACGGTCTATG | [1] |
| *mcr-1*-qR | GCACACCCAAACCAATGATAC |  |
| *amiA*-qF | AATGGCGAAATACCTGTCTG | this study |
| *amiA*-qR | GTTGCGGCTGTGCAGTTTA |  |
| *pmrB*-qF | ACGCTGGATAACGAAAGG |  |
| *pmrB*-qR | GCCACTAACGGTGCTACAT |  |
| *arnT*-qF | TTGCCTTTCCACGTCTGTG |  |
| *arnT*-qR | GGTGACCTGGTTTACTCTGC |  |
| *eptA*-qF | F:AAATCAAACCTGCCACCTC |  |
| *eptA*-qR | R:GTTATTGCGGAACAACGAG |  |
| *cpxA*-qF | CCGTAATGGTGATACCGTCTT |  |
| *cpxA*-qR | GAGCAAATGGGCAAGTCG |  |
| *fumB*-qF | CGCAATATGATACGGAGGA |  |
| *fumB*-qR | GTGGTTCGGCAAACAAGAC |  |
| *gltA*-qF | CCAGCGAGTCGTGATAGAA |  |
| *gltA*-qR | ATCCTGCTGAATGGTGAAA |  |
| *sdhA*-qF | TCTTCGCCTTTCTCGTTCA |  |
| *sdhA*-qR | CTCCCGTACCTTCGCTCAC |  |
| *SdhC*-qF | GACGACGTGATACGCCAGA |  |
| *SdhC*-qR | GCGATAGCGTCCATTCTCC |  |
| *flhC*-qF | ATGGCGGTTGACATAAGC |  |
| *flhC*-qR | AGTGCCCACAAGCAGAAG |  |
| *Fimh*-qF | CCGAAGTTCCTACTGCTCC |  |
| *Fimh*-qR | ATTCCTCTTACCGTTTATTGTG |  |
| MCR-1Ser284A-F | TCACATCGTGCGGCACAGCGACGGCGTATTCTGTG | [2] |
| MCR-1Ser284A-R | ACGCCGTCGCTGTGCCGCACGATGTGACATTGCTAAA |  |
| *16S rRNA-q*F | CCTCAGCACATTGACGTTAC | [3] |
| *16S rRNA-q*R | TTCCTCCAGATCTCTACGCA |  |

[1] S. Bontron, L. Poirel, P. Nordmann, Real-time PCR for detection of plasmid-mediated polymyxin resistance (*mcr-1*) from cultured bacteria and stools, Journal of Antimicrobial Chemotherapy, 71 (8) (2016) 2318-20.

[2] Sheng Q, Hou X, Wang Y, Wang N, Deng X, Wen Z, Li D, Li L, Zhou Y, Wang J. Naringenin Microsphere as a Novel Adjuvant Reverses Colistin Resistance via Various Strategies against Multidrug-Resistant Klebsiella pneumoniae Infection. J Agric Food Chem. 2022 Dec 28;70(51):16201-16217. doi: 10.1021/acs.jafc.2c06615. Epub 2022 Dec 19. PMID: 36530172.

[3] Y. Zhai, H. Huang, J. Liu, H. Sun, D. He, Y. Pan, G. Hu, CpxR overexpression increases the susceptibility of *acrB* and *cpxR* double-deleted *Salmonella enterica serovar* Typhimurium to colistin, Journal of Antimicrobial Chemotherapy. 73 (11) (2018) 3016-3024.

**Table S4 MIC values of colistin alone or in combination with piperine in *E. coli* EC15 treated with different cations. Repeat the data three times**

| Species | cations | MIC (μg/mL)  Colistin alone | MIC (μg/mL) in combination  Piperine (32μg/mL)+COL | FIC Index |
| --- | --- | --- | --- | --- |
| *E. coli* EC15 | - | 8 | 0.5 | 0.156 |
|  | +50μg/mL Zn^2+^ | 8 | 1 | 0.313 |
|  | +50μg/mL Ca^2+^ | 8 | 4 | 0.375 |
|  | +50μg/mL Mg^2+^ | 8 | 2 | 0.313 |
|  | +50μg/mL K^+^ | 8 | 1 | 0.188 |
|  | +50μg/mL Na^+^ | 8 | 0.5 | 0.125 |
|  | + 0.1 mM EDTA | 4 | 0.25 | 0.125 |
|  | + 1 mM EDTA | 2 | 0.0625 | 0.094 |
|  | + 10% serum | 8 | 1 | 0.188 |
|  | + 10% DMEM | 8 | 1 | 0.188 |
|  | +100μM LPS | 16 | 4 | 0.375 |

**Table S5 The FICI of piperine with colistin against MCR-1 wild and mutant strains in *E. coli***

| Strains | Antibiotic | MIC | | FICI |
| --- | --- | --- | --- | --- |
|  |  | alone | Conbination with  PIP (32 μg/mL) |  |
| MCR-1-WT | colistin | 4 | 0.5 | 0.188 |
| MCR-1-Ser284Ala | colistin | 4 | 1 | 0.313 |

**Supplementary Figures**


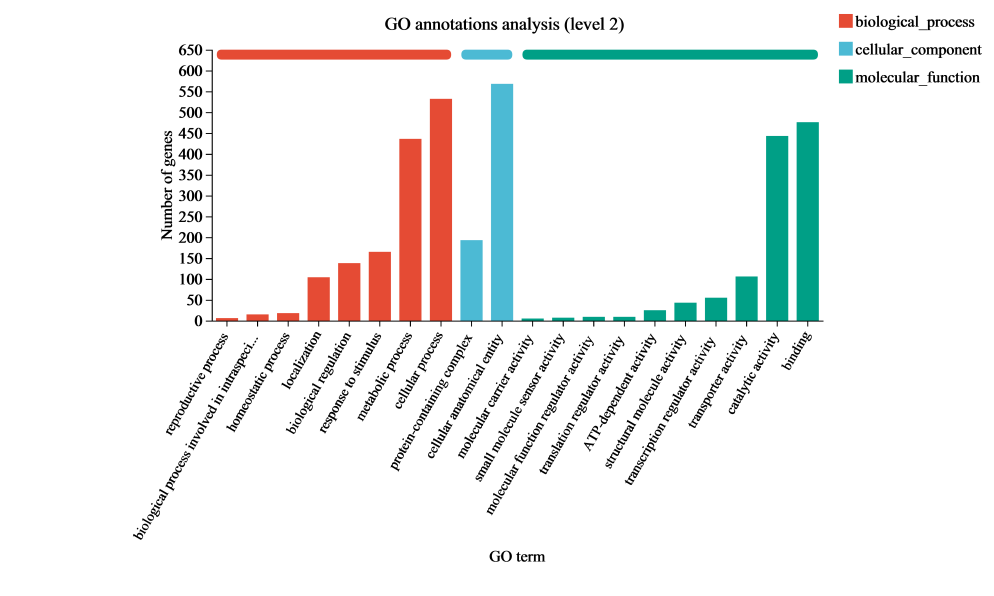


**Fig. S1 Transcriptomic evaluation of piperine combined with colistin in the treatment of *E. coli* EC15, and GO enrichment analysis of DEGs.**


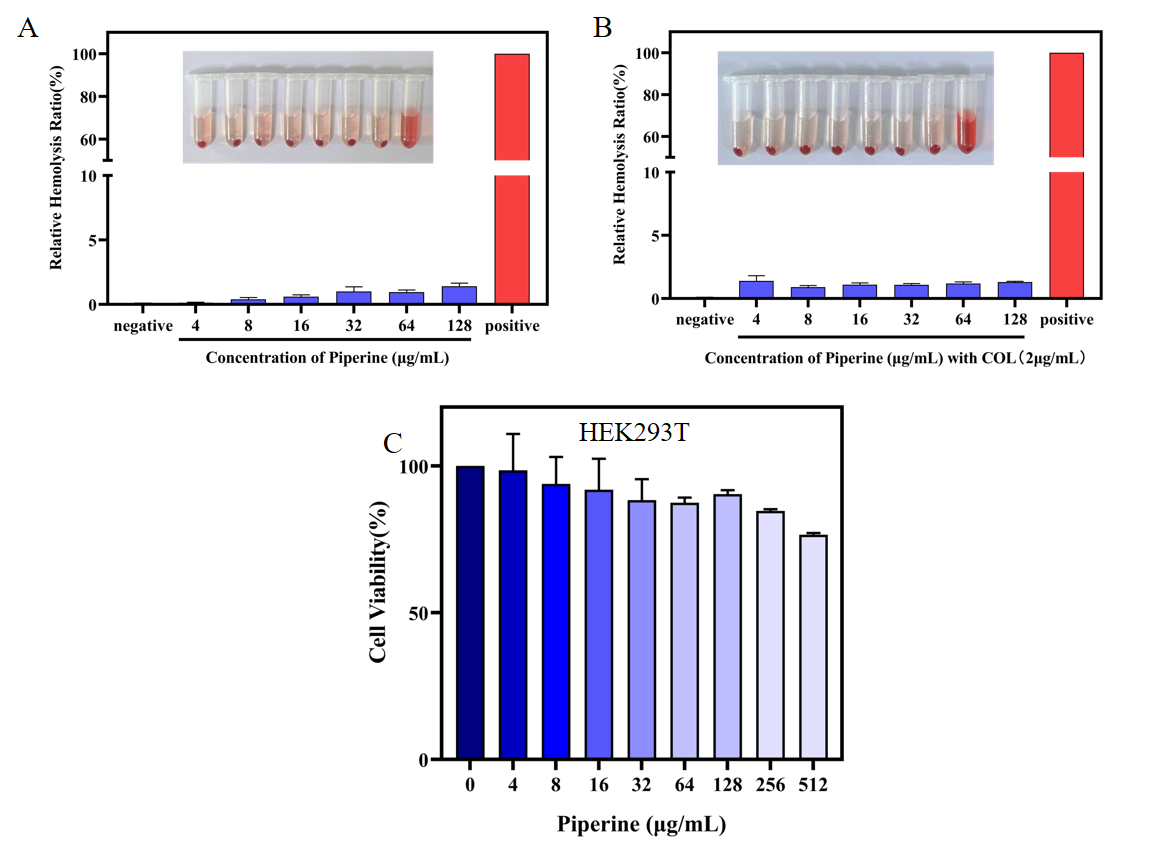


**Fig. S2 Piperine demonstrated low hemolysis and cytotoxicity.** (A, B) Hemolysis rate of mouse red blood cell exposed to piperine alone or the combination of piperine

and colistin. (C) Cytotoxicity of piperine in HEK293T cells.

**
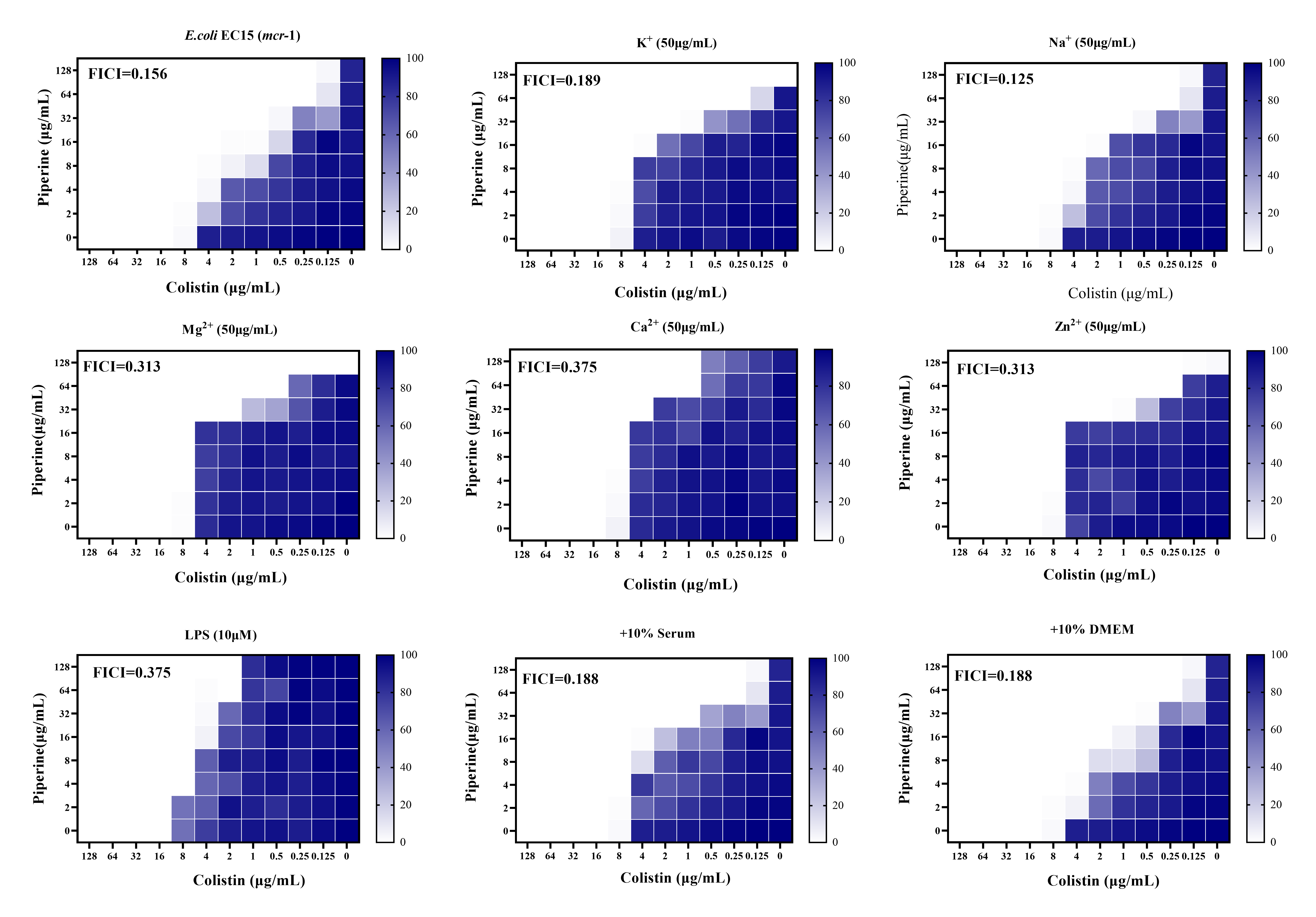
**

Fig. S3. The influence of metal ions, LPS, 10% serum and 10% DMEM to the synergy of piperine and colistin, related to Table S4. Dark blue regions represent higher cell density. Data represent the mean OD (600 nm) of two biological replicates.


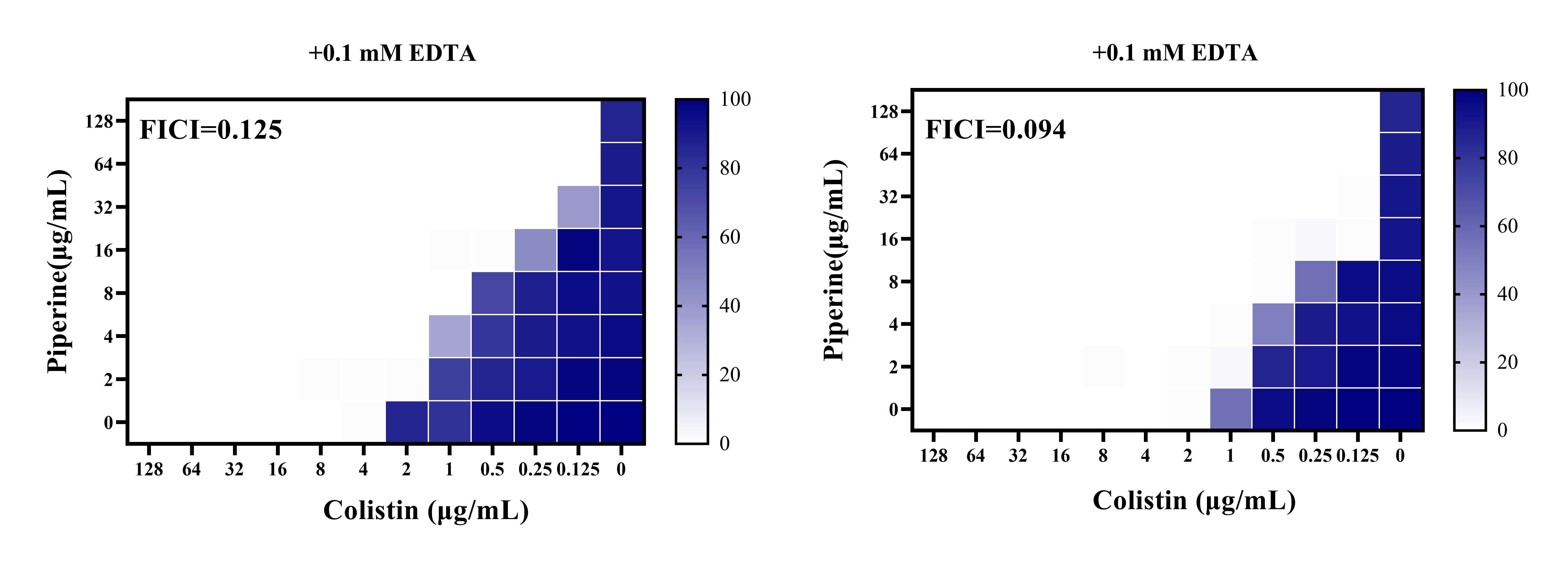


Fig. S4. Effect of EDTA on the synergistic activity between piperine and colistin against E. coli EC15. Dark blue regions represent higher cell density. Data represent the mean OD (600 nm) of two biological replicates.
